# Investigating the influence of ubiquinone blood level on the abilities of children with specific learning disorder

**DOI:** 10.1186/s41983-018-0029-8

**Published:** 2018-11-27

**Authors:** Ehab Ragaa Abdelraouf, Ayman Kilany, Adel F. Hashish, Ola Hosny Gebril, Suzette Ibrahim Helal, Haytham Mohamad Hasan, Neveen Hassan Nashaat

**Affiliations:** 1 Research on Children with Special Needs Department, Medical Research division, National Research Centre, Elbuhouth Street, 12622, Dokki, Cairo, Egypt; 2 Learning Disability Research Clinic, Medical Research Centre of Excellence, National Research Centre, Cairo, Egypt; 3 Pediatric Neurology Research Clinic, Medical Research Centre of Excellence, National Research Centre, Cairo, Egypt; 4 Neurology Research Clinic, Medical Research Centre of Excellence, National Research Centre, Cairo, Egypt; 5 Neuropsychiatry Department, Faculty of Medicine, Helwan University, Cairo, Egypt; 6 Phoniatric Research Clinic, Medical Research Centre of Excellence, National Research Centre, Cairo, Egypt

**Keywords:** Specific learning disorder, Ubiquinone level, Correlation, Memory

## Abstract

**Background:**

Ubiquinone has antioxidant properties and has been linked to cognitive performance in some neuropsychiatric disorders. Its role in specific learning disorder manifestations has not been previously investigated. Therefore, the aim of this study was to measure the blood levels of ubiquinone in a group of children with specific learning disorder in comparison to typically developing children and to investigate the correlation between ubiquinone levels in children with specific learning disorder and some of their intellectual capabilities, reading, spelling and writing performance.

**Methods:**

The study included 71 native Arabic speaking children: 31 in the specific learning disorder group and 40 in the typically developing (TD) group. The abilities of the children with specific learning disorder were evaluated by the Stanford-Binet Intelligence Scale-4th edition, the Dyslexia Assessment Test, and the Illinois Test of Psycholinguistic Abilities. The level of ubiquinone was measured in both groups by ELISA. Correlation between some aptitudes of children with specific learning disorder and the ubiquinone level was performed.

**Results:**

The blood levels of ubiquinone in the children with specific learning disorder group were less than those in the TD group. Correlation analysis revealed a significant positive correlation between ubiquinone and the scores of backward digit span abilities.

**Conclusions:**

Ubiquinone has a role in the auditory working memory performance of children with specific learning disorder (with impairment in reading). The decreased levels of ubiquinone in this sample of children with specific learning disorder could have participated in the pathogenesis of this disorder.

## Key messages

This is the first study that was addressed to verify the involvement of ubiquinone in specific learning disorder. Ubiquinone is suggested to have a role in auditory working memory performance of children with specific learning disorder.

## Background

The ubiquinone (coenzyme q 10) is an important factor in the mitochondrial respiratory chain and it has antioxidant properties. It has a neuroprotective effect and increases cerebral energy metabolism [1]. Therefore, it is suggested to be involved in the proper functioning of the central nervous system, auditory sensory cells, auditory nerve, retina, skeletal muscles, and endocrine glands. These systems are highly dependent on oxidative metabolism for energy generation. All these systems have essential roles in learning and share in cognitive development [2]. Some studies suggested that ubiquinone supplementation could have a protective role on cognitive performance in animal and human trials and that its level in the brain could be related to cognitive performance [3–5].

Specific learning disorder is a developmental disorder that impedes the ability to learn or use specific academic skills such as reading, writing, or arithmetic skills which are the foundation for other academic learning [6]. Children with specific learning disorder frequently manifest memory deficits, word finding difficulties, and reading and writing disorders [7, 8]. Many theories have been proposed to explain these deficits that are evident despite the lack of intellectual disability in such children. Neuroanatomical variations and auditory and visual processing deficits have been suggested to explain the difficulties which are manifested by the children with learning disorder. These neuroanatomical deficits were variable among studies [9–11]. Therefore, identifying the role of measurable biomarkers could suggest a possible pathogenesis and would help in proper management and early detection of such mysterious disorder.

The aim of this study was to measure the blood level of ubiquinone in a group of children with specific learning disorder in comparison to a group of typically developing ones and to investigate the correlation between blood levels of learning disorder children and their intellectual capabilities, reading, spelling, and writing performance.

## Methods

This comparative case-control study included 71 native Arabic speaking participants divided into two groups. Group I included 31 children with learning disorders (age 8.3 ± 1.1; 22 males, 9 females; IQ range 81–115). They visited the Learning Disability Research Clinic, the Phoniatric Research Clinic, the Psychiatry Clinic, and the Pediatric Neurology Research Clinic in the Medical Research Centre of Excellence in the National Research Centre (from January to December 2017) complaining of poor scholastic achievement. They were selected when their age ranged from 6 to 10 years and when they are enrolled in the education system and if they were given the opportunity to study well yet fail to cope. They were included in the study when matching the criteria of specific learning disorder according to the Diagnostic and Statistical Manual of Mental Disorders-5 through psychiatric interview performed by an expert psychiatrist [6]. Children who manifested major neurological, motor, MRI, or EEG abnormalities, sensory deficits, and comorbid psychiatric disorders or obtained an IQ below 70 were excluded from the study. Group II included 40 typically developing children. They were among the relatives of children visiting the above mentioned clinics. They were selected when their age ranged from 6 to 10 years, their linguistic abilities matched their age or were beyond, and their scholastic achievement was good without the need of extra support. They volunteered to share in the study. If they had a history of developmental neuropsychiatric disorders, they were excluded. They matched the patients group for age, sex, and socioeconomic status (age 8.05 ± 1.15; 28 males, 12 females; IQ range 92–117). They all were enrolled in the national education system. Written informed consents were obtained from parents of participants. The study was approved by the medical research ethics committee of the National Research Centre.

All participants were subjected to the following:

- Full personal and medical history with thorough clinical examination.
- Mini International Neuropsychiatric Interview for Children (M.I.N.I. Kid) which is a short structured diagnostic interview for DSM-IV and ICD-10 psychiatric disorders [12]. It was used to exclude other psychiatric comorbidities in the patient group and to exclude psychiatric disorders in the control group. The Arabic version was validated and it has good reliability and validity. It is used in many studies in Arab countries [13].
- The Arabic version of Stanford-Binet Intelligence Scale, 4th edition (SB-IV). It was used for intelligence quotient (IQ) assessment. It has four sub-items including verbal reasoning, abstract/visual reasoning, quantitative reasoning, and short-term memory. The verbal reasoning evaluates verbal knowledge and the ability to apply verbal skills to new situations. The abstract/visual reasoning reflects the ability to visualize patterns, visual/motor skills, problem-solving skills, and mathematic operations through the use of reasoning. The quantitative reasoning measures numerical reasoning, concentration, knowledge, and application of numerical concepts. The short-term memory assesses concentration skills, short-term memory, and sequencing skills of auditory and visual tasks [14, 15].

The group of specific learning disorder was further assessed by the following:

- The Arabic version of Dyslexia Assessment Test (DAT). It was used for evaluating the reading, spelling, writing, and some other intellectual abilities of the participants. It has the following subtests: rapid naming, bead threading, 1-min reading, postural stability, phonemic segmentation, 2-min spelling, backward digit span, nonsense passage reading, 1-min writing, verbal fluency, and semantic fluency [16, 17]. The raw scores of the subtests were used to determine the quality of the performance guided by tables for each certain age group. An at-risk quotient is finally obtained. This quotient increases with worse performance in the subtests. The quality of performance in the subtests was transformed into a grading system from 1 to 5 for correlation purposes being higher with better performance.
- The Arabic version of the Illinois Test of Psycholinguistic Abilities (ITPA). It has the following subtests: auditory reception, visual reception, auditory association, visual association, verbal expression, manual expression, grammatic closure, visual closure, auditory sequential memory, and visual sequential memory [18, 19]. The test was applied for obtaining raw scores for each child in each subtest. The raw scores of each subtest were converted to scaled scores according to the child’s mental age. These scores reflect the child’s performance in the subtests. The sum of the scaled scores was used to obtain a mean of scaled scores for each child.

### Biochemical measures

Blood samples from both groups were obtained and analyzed for estimating the levels of ubiquinone which were evaluated by the enzyme-linked immunosorbent assay (ELISA) [20].

### Analysis of data

Data were analyzed using SPSS computer package version 17 (SPSS, Chicago, IL, USA). The independent t test was used for comparison between the specific learning disorder group and the typically developing (TD) children regarding the ubiquinone levels. Spearman’s correlation coefficient was used to investigate the correlation between the measured ubiquinone levels and the scores of evaluated aptitudes of the Dyslexia Assessment Test. When *p* for any item was less than 0.05, it was considered to be statistically significant.

## Results

The total IQ scores for the specific learning disorder group and the TD group were 94.2 ± 7 and 100 ± 6.5 respectively. In the specific learning disorder group, the mean of the IQ scores of the subtests of SB-IV were above 90°. Most of the scaled scores of the subtests of the ITPA were above 30 (Table 1). All participants in this group manifested specific learning disorder with impairment in reading. The at-risk quotient and the mean of the scaled scores of the ITPA scores were as follows: 1.6 ± 0.6; 34.3 ± 4. The mean level of ubiquinone in the learning disorder group was 0.753 ± 0.38 (range 0. 23–1.47) while it was 1.357 ± 0.52 (range 0.52–2.31) in the TD group. Comparison between the groups regarding ubiquinone levels showed significant statistical difference being less in the specific learning disorder group (*t* = 3.8; *p* = 0.0003).

**Table 1:** Mean and standard deviation of IQ scores, subtests of SB-IV, and the scaled scores Illinois Test of Psycholinguistic Abilities subtests for the specific learning disorder group

Item	Scores mean	Standard deviation
Verbal reasoning	95	12
Abstract/visual reasoning	94	14
Quantitative reasoning	94	10
Working memory	90	15
Total IQ	94.2	7
Auditory reception	31.3	10
Visual reception	31.6	6
Auditory association	33.6	6
Visual association	35.8	7
Verbal expression	33.4	3
Manual expression	36.3	4
Grammatic closure	43.4	6
Visual closure	29.6	7
Auditory sequential memory	29.6	6
Visual sequential memory	29.6	8
Mean of scaled scores of Illinois test	34.3	4

Correlation analysis revealed that the ubiquinone levels showed statistically significant positive correlation with backward digit span subtest of the DAT (*r* = 0.4; *p* = 0.04). Other abilities were not correlated with the ubiquinone levels (Table 2).

**Table 2:** Correlation analysis between ubiquinone levels and the Dyslexia Assessment Test subtests and its at-risk quotient in the specific learning disorder group

Items	*R* value	*P* value
Rapid naming	− 0.03	0.8
Bead threading	− 0.2	0.1
One-minute reading	0.1	0.6
Posture stability	0.04	0.8
Phonemic segmentation	0.08	0.6
Two-minute spelling	0.1	0.6
Backward digit span	0.4	0.04^a^
Nonsense passage reading	0.06	0.7
One-minute writing	0.1	0.3
Verbal fluency	0.1	0.5
Semantic fluency	0.1	0.3
At-risk quotient	− 0.1	0.5

^a^Significant

## Discussion

The mean IQ scores and most of the scaled scores of the ITPA subtests were within normal range in the group of children with specific learning disorder in the present study. Most of their intellectual abilities required for learning are intact. However, they fail to cope with school requirements concerning reading, spelling, and other aptitudes. Therefore, exploring possible factors that could influence their learning capabilities is of special importance.

Disruption of inhibitory signaling within cortical circuits is a central feature of cognitive disabilities across several neuropsychiatric disorders [21]. Learning disorder was reported to be associated with variations in the structure and function of some brain areas sharing in reading performance. Further, derangements in the connections between these areas were found. These areas include left temporoparietal region and the left inferior frontal gyrus with abnormalities in the arcuate fasciculus in-between [22].

Considering that brain consumes huge amount of oxygen, it produces a lot of reactive oxygen species. Therefore, brain tissue is sensitive to any imbalance of oxidant-antioxidant system. The ubiquinone is an antioxidant that has a neuroprotective value [23]. It has a protective role on polyunsaturated fatty acids which constitute an important component of neurons and myelin sheath [24]. The learning disorder participants manifested less ubiquinone levels in relation to controls which could indicate oxidative stress. Therefore, a decrease in its level could have adverse influence on brain functioning and connections required between different areas responsible for learning which could lead to learning disorder. Furthermore, ubiquinone was found to enhance the phosphatidylinositol 3-kinase pathway. This pathway has been suggested to play an important role in protecting the neural stem cells and controlling neuronal cell survival. It is highly involved in human neurogenesis needed for memory performance [25]. Additionally, ubiquinone was implicated in motivation and desire to engage in activities [26]. The lack of motivation and the reduced engaging in activities have been noticed in some children with learning disorder which could further magnify the learning difficulties in such children [27].

The forms of long-term memory including declarative, episodic, or spatial memory were related to the hippocampus located in the medial temporal lobe. Moreover, the prefrontal cortex has been implicated in working memory and long-term memory performance and planning of complex cognitive behavior [28]. Proper memory performance is a prerequisite for learning. Moreover, performance in working memory influenced the spelling abilities of dyslexic children [8]. The ubiquinone levels in the specific learning disorder group were positively correlated with backward digit span scores which represents auditory working memory.

Aboul-Fotouh [26] reported that ubiquinone supplementation improved frontal lobe and hippocampal mitochondrial respiratory chain complexes and creatine kinase responsible for energy production in these brain areas. These findings suggest a link between ubiquinone and hippocampus and prefrontal cortex functioning. Hence, the correlation between ubiquinone level and the backward digit span representing memory could stem from the links between ubiquinone, hippocampus, and the prefrontal cortex. The lack of direct relation between ubiquinone and reading or spelling abilities of the participants could be attributed to the complexity of these skills and the interference of many factors in the child’s performance in such skills. This study, to our knowledge, is the first one investigating the correlation between ubiquinone level and the different aptitudes of children with specific learning disorder, thus highlighting the relation in-between these measures.

## Conclusions

Ubiquinone has a role in the auditory working memory performance of children with specific learning disorder with impairment in reading. The decreased levels of ubiquinone in this sample of children with learning disorder could have participated in the pathogenesis of specific learning disorder.

## Abbreviations

DAT: Dyslexia Assessment Test
DSM-5: Diagnostic and Statistical Manual of Mental Disorders, Fifth Edition
DSM-IV: Diagnostic and Statistical Manual of Mental Disorders, Fourth Edition
EEG: Electroencephalogram
ELISA: Enzyme-linked immunosorbent assay
ICD-10: International Statistical Classification of Diseases and Related Health Problems 10th Revision
IQ: Intelligence quotient
ITPA: Illinois Test of Psycholinguistic Abilities
M.I.N.I. Kid: Mini International Neuropsychiatric Interview for Children
SB-IV: Stanford-Binet Intelligence Scale, 4th edition
TD: Typically developing
